# BowSaw: Inferring Higher-Order Trait Interactions Associated With Complex Biological Phenotypes

**DOI:** 10.3389/fmolb.2021.663532

**Published:** 2021-06-17

**Authors:** Demetrius DiMucci, Mark Kon, Daniel Segrè

**Affiliations:** ^1^ Bioinformatics Graduate Program, Boston University, Boston, MA, United States; ^2^ Biological Design Center, Boston University, Boston, MA, United States; ^3^ Department of Mathematics and Statistics, Boston University, Boston, MA, United States; ^4^ Department of Biology, Boston University, Boston, MA, United States; ^5^ Department of Biomedical Engineering, Boston University, Boston, MA, United States; ^6^ Department of Physics, Boston University, Boston, MA, United States

**Keywords:** high-order interactions, microbiome, epistasis, random forest, Boolean rules, decision tree, complex phenotypes

## Abstract

Machine learning is helping the interpretation of biological complexity by enabling the inference and classification of cellular, organismal and ecological phenotypes based on large datasets, e.g., from genomic, transcriptomic and metagenomic analyses. A number of available algorithms can help search these datasets to uncover patterns associated with specific traits, including disease-related attributes. While, in many instances, treating an algorithm as a black box is sufficient, it is interesting to pursue an enhanced understanding of how system variables end up contributing to a specific output, as an avenue toward new mechanistic insight. Here we address this challenge through a suite of algorithms, named BowSaw, which takes advantage of the structure of a trained random forest algorithm to identify combinations of variables (“rules”) frequently used for classification. We first apply BowSaw to a simulated dataset and show that the algorithm can accurately recover the sets of variables used to generate the phenotypes through complex Boolean rules, even under challenging noise levels. We next apply our method to data from the integrative Human Microbiome Project and find previously unreported high-order combinations of microbial taxa putatively associated with Crohn’s disease. By leveraging the structure of trees within a random forest, BowSaw provides a new way of using decision trees to generate testable biological hypotheses.

## Introduction

The production of large biological data sets with high-throughput techniques has increased the utilization of supervised machine learning algorithms (Goodswen et al., 2021; Reel et al., 2021), including support vector machines (Yang et al., 2021), neural networks (Rampelli et al., 2021) and random forests (Dicker et al., 2021), to produce predictions of complex phenotypes (e.g., healthy vs. disease) from measurable traits (Cesario et al., 2021; Hughes et al., 2021; Marcos-Zambrano et al., 2021). These algorithms use measurements of relevant traits such as gene variants, the presence/absence of microbial taxa, or metabolic consumption variables as predictors. Categorical prediction of phenotypes is typically the end goal of these applications. However, an additional benefit of these algorithms is the potential to extract explanatory classification rules. In this context, a rule is defined as a Boolean function of a set of traits, such that the value of the function is 1 (true) when the traits are associated with a given phenotype. Identifying the relationships between the traits involved in classification rules may yield key insights into the biological processes associated with important phenotypes (Furqan and Siyal, 2016; Visscher et al., 2017). This realization is creating demand for methods that assist in the interpretation of supervised machine learning methods (Azmi et al., 2019; Nguyen et al., 2019; Le et al., 2020), especially when the measured traits may be causal agents of disease states, such as genetic variants or microbial taxa (LaPierre et al., 2019). Identifying classification rules associated with a phenotype of interest is valuable because these rules are likely to carry information about the causal mechanisms that generate the phenotype.

Algorithms that are particularly valuable in this respect are those involving decision trees, such as random forests, since decision trees are easily interpretable (Brodley and Friedl, 1997). Decision trees are rule-based classifiers, where rules arise from a series of “yes-no” questions that can efficiently divide the data into categorical groups. In a biological context, such rules may arise from sets of genes whose simultaneous modulation could affect a phenotype, or sets of microbial species whose co-occurrence may be associated with a disease state. While in several cases it seems like disease phenotypes are uniquely associated with a single specific pattern [e.g., retinoblastoma (Knudson, 1971)], there is increasing evidence for cases in which multiple distinct patterns can be associated with (and potentially causing) the same high-level phenotype (Emily et al., 2009; Leem et al., 2014). A particular example we will explore in this work is the multiplicity of distinct microbial presence/absence patterns which may be associated with Crohn’s disease (Proctor et al., 2019). Crohn’s disease has five clinically defined sub-types (Reading, 2014) but studies of the associated microbiome do not usually indicate which form of Crohn’s disease a donor has been diagnosed with. Each sub-type of the disease may be associated with different microbes, each requiring different treatment regimes. As discussed later, we hypothesize that the different rules associated with a given phenotype label may be related to these different subtypes, with potential therapeutic implications.

**FIGURE 1:**
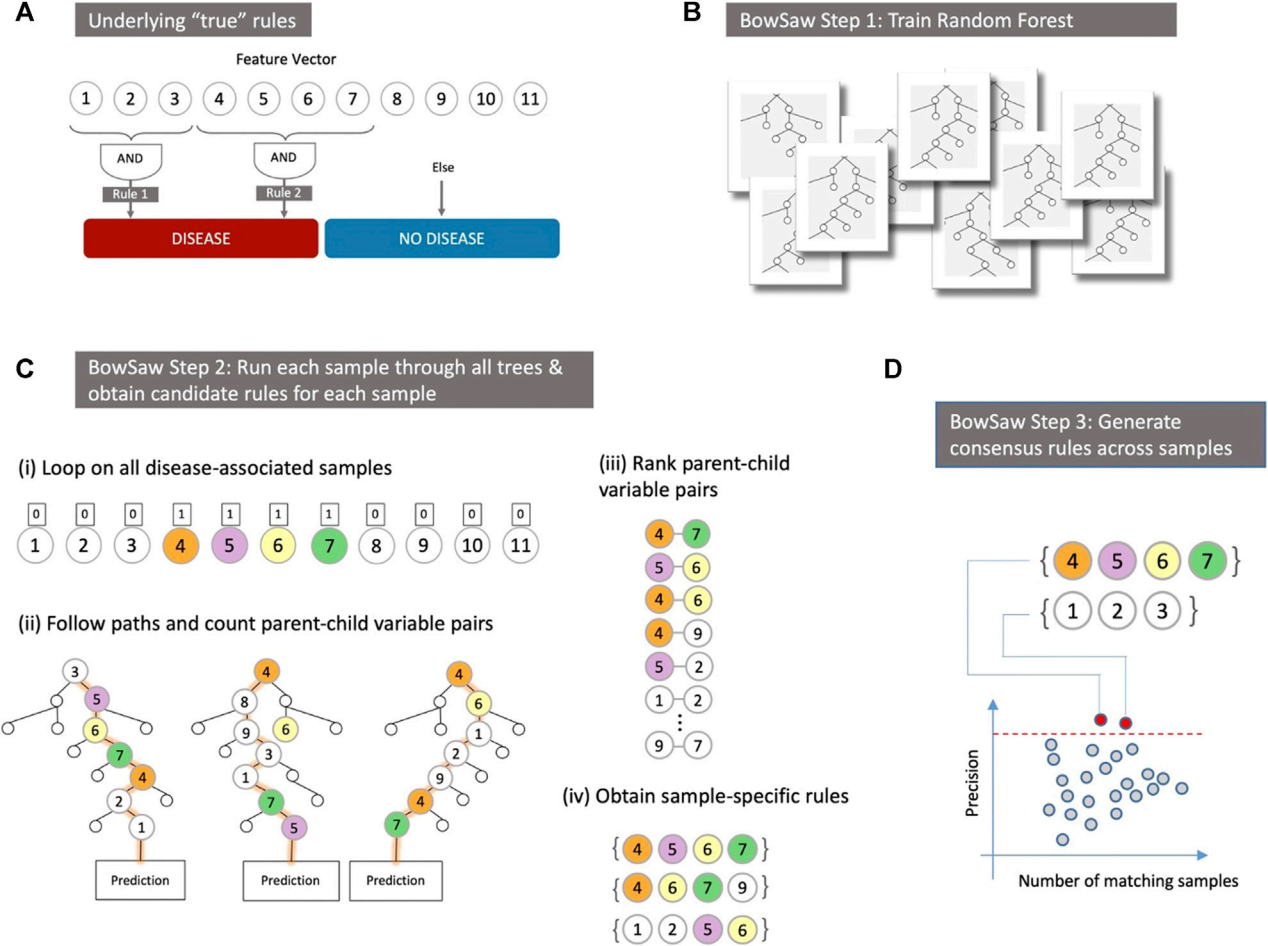
**(A)** In a hypothetical dataset there are two phenotype labels–“Disease” and “No Disease” that we wish to discriminate based on input predictor variables. In this example, there are two distinct high-order patterns that both confer the same “Disease” phenotype. Our goal is to identify a potentially diverse set of patterns (or, in this simplified case, all patterns) that are associated with the “Disease” label. **(B)** Instead of exhaustively evaluating variable combinations we leverage the structure that emerges from an ensemble of decisions trees like those produced by a trained random forest. **(C)** For each sample with the observed phenotype “Disease” we first identify the vector containing its input values (i). Then follow the paths it takes downs each tree that attempts to predict its class and record the frequency of parent-child variable pairs (ii). Next, we rank parent-child variable pairs in descending order of frequency (iii). Finally, we use a great search to construct a sample-specific rule that is fully associated with the “Disease” phenotype (iv). **(D)** All sample-specific rules are evaluated in order to obtain a consensus set of rules that combined account for all samples with the “Disease” phenotype.

The fact that there may be multiple etiologies that generate the same or similar phenotypes complicates the straightforward interpretation of parameter coefficients or variable importance scores (Louppe, 2014; Wright et al., 2016). Uncovering the multiple interactions between predictive variables as they relate to phenotypic labels remains a challenging statistical endeavor, but one that is of paramount importance. In an ideal situation, one could conduct a best subset search, evaluating all possible classification rules that can be defined using the data and identifying a set of rules that concisely explain the observed associations. This strategy is computationally intractable using a brute force approach: even a relatively small biological data set of 50 features with binary coding would require examining over 2^50^ variable sets and many more specific rules (since the specific value of features, 0, 1, or ‘omitted’, is important). Identifying the associated rules that a random forest uses to classify a given sample (a specific row of the data matrix) offers the possibility to bypass the brute force approach and enables the development of mechanistic hypotheses for follow-up studies. This challenge, and an overview of the key strategy we propose, are illustrated in Figure 1. In Figure 1A we depict a toy model where measured variables (traits) have only two possible values (e.g., present/absent), the high-level phenotype (category) is binary (e.g., no disease/disease), and two distinct Boolean rules can both generate the phenotype. The goal in this case is to identify each of the rules that are associated with the phenotype. The multiple Boolean rules obtained in this manner can be thought of as a consensus decision tree that possesses the most informative branches of the forest with respect to a given class label. In this work, we will show how this can be achieved by in-depth analyses of any given random forest (RF) (Figure 1B).

The random forest algorithm intrinsically takes advantage of non-linear relationships between variables and is widely used in the life sciences (Boulesteix et al., 2012; Nguyen et al., 2013; Touw et al., 2013). RFs, when used to distinguish between disease states known to have multiple causes, often result in excellent classifiers (Duvallet et al., 2017; Franzosa et al., 2019). It has also been reported that RFs capture subtle statistical interactions between variables (Louppe, 2014). Unfortunately, an RF is not straightforwardly interpretable despite its hierarchical structure, and recovering those interactions is notoriously difficult (Wright et al., 2016) due in large part to the method’s reliance on ensembles of trees (Breiman, 2001). The difficulties in interpretation created by these properties has led many to refer to RF as a ‘black box’ model (Castelvecchi, 2016).

Identifying the rules that a RF utilizes in classification tasks is an active area of research, and many strategies have been developed to address this problem. Effective strategies have focused on evaluating how individual variables influence the classification probabilities of specific samples (Palczewska et al., 2013; Welling et al., 2016), pruning existing decision rules found in the tree ensemble to produce compact models (Deng, 2019), computing conditional importance scores (Strobl et al., 2008), or iteratively enriching the most prevalent variable co-occurrences through regularization (Basu et al., 2018). These approaches offer valuable methods for the identification of statistical interactions between variables. However, we and others have observed that while these methods are capable of recovering a true causal rule in simulated data when exactly one such rule is present, the existence of multiple rules associated with one phenotype can confound interpretation efforts (Basu et al., 2018).

Here we describe BowSaw, a new set of algorithms that utilizes variable interactions in a trained RF model in order to extract multiple candidate explanatory rules. With BowSaw, we set out to develop a *post hoc* method intended to aid in the discovery of these rules when the input variables are categorical in nature. The primary approach of BowSaw is to start by approximating a best combination of variables (i.e., a rule) that explain the forest’s predictions for individual samples of a given class in the data set and then to curate the collection of best combinations to obtain a concise set of combinations that collectively segregate a class of interest with high precision. For individual samples a rule is identified by systematically quantifying the co-occurrence of specific variable pairs across trees in the forest that attempt to predict the class of the sample (out-of-bag trees) and then using the frequency of these co-occurring variable pairs to guide the construction of a rule that precisely identifies the sample as its observed class. For the entire set of samples, we then curate the collection of all rules identified in this way, in order to produce a small set of rules that are broadly and precisely applicable to samples of the given class label.

We first demonstrate that BowSaw can recover true rules (when they exist) by applying the algorithms to simulated data sets of varying complexity. We then apply BowSaw to a study on the role of the gut microbiome on Crohn’s disease (Proctor et al., 2019), and show that it can find a previously unreported combination of microbial taxa that is broadly and precisely associated with Crohn’s disease samples in the data set. In its current implementation, BowSaw can be applied to any dataset with categorical or discrete predictors with any number of class labels.

## Methods

### Overview of the Pipeline

Provided with a trained random forest and a training set, BowSaw goes through three steps in order to generate a candidate rule (variable-value combination) for each sample associated with the phenotype of interest. First, for a specific sample, the *Count* algorithm counts the frequency of unique ordered pairs of variables encountered along each of its out-of-bag trees in the forest (Figure 1C–step 2). Second, for that sample, the *Construct* algorithm takes the counts from the first step and generates a list of ordered pairs, ranked by their frequencies, then uses this list as a guide to construct a candidate decision rule (which could consist of two or more variables) that is associated with the observed phenotype at a user defined precision threshold (Figure 1C–steps 3–4). Finally, the *Curate* algorithm pools the candidate decision rules from each sample together and greedily selects a subset of rules that collectively account for all of the samples with the desired phenotype (Figure 1D). Optionally, the *Sub-rule* algorithm can be used to generate pruned versions of candidate rules prior to applying the Curate algorithm in order to obtain a more concise, albeit less specific, set of candidate rules. The Count and Construct algorithms generate the candidate rules for individual samples while the Curate and Sub-rule algorithms produce a combined set of rules that account for all samples with the chosen phenotype.

In the following section, we provide a description of the inputs BowSaw takes and the algorithms that implement these steps along with pseudocode.

### Inputs

BowSaw takes as inputs a dataset, ***D***, composed of N observed vectors $x_{i}$ (together with their respective classes $k_{i}$) each of p categorical variables. There are assumed to be K possible class labels for each vector in ***D*** which for the purposes of this discussion denote different phenotypes. A random forest is assumed to be trained on ***D*** to distinguish the classes k=1,…,K *.* Additionally, BowSaw takes as input the feature vector $x_{i}$ of a specific sample for which the goal is to identify a set of simplified rules associated with the phenotype $k_{i}$.

### Counting Stubs

Given an RF machine ***M*** trained on dataset ***D*** and a feature vector $x=\left(x_{1},x_{2},\ldots ,x_{p}\right)\in D$ **,** the first sub-routine of our method (the *count algorithm*) proceeds as follows. It starts by identifying among the set of trees in ***M***, those sub-paths (sequences of successive variable indices) encountered by sample x as it travels through $M_{x}$, its set of out-of-bag trees. An out-of-bag tree is a tree for which x was not included in the training set. For a specific path ***P*** in $M_{x}$ the sequence of successive variable indices forms a vector $v=(v_{1},\ldots ,v_{r}$) (note that each $v_{j}$ is one of the variables $x_{j}$). Each stub (ordered pair of sequentially encountered variables $v_{i}v_{i+1}$) in all out-of-bag elements along P for *i =* 1, … *r*-1 is accounted for in a p×p matrix $C^{x}$, where the element $C_{ij}^{x}$ records the number of stubs containing the ordered pair of variables $x_{i}$ and $x_{j}$ among all paths of $M_{x}$. We restrict the counting to sequentially encountered variables because higher order interactions involving 3 or more sequential variables are much rarer and would require many more trees than is necessary to build an acceptable classifier.

Algorithm 1

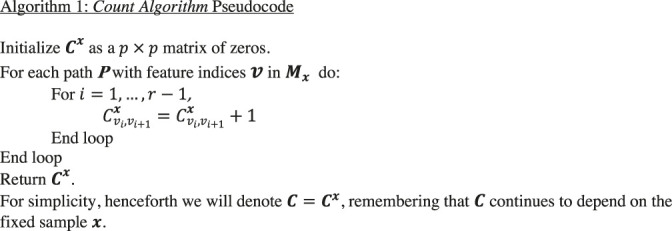

### Constructing a Candidate Rule

A *rule* for classifying to a test point x will have the form “If $x_{I}=a_{I}$ then classify x to class k”. Here I is a designated subcollection of the variable indices i=1,…,p, and $x_{I}=\left(x_{i_{1}},\ldots ,x_{i_{\left|I\right|}}\right)$ is the sub-vector of current vector $x=\left(x_{1},\ldots ,x_{p}\right)$ corresponding just to the indices $i_{j}\in I$. The vector $a_{I}=\left(a_{i_{1}},\ldots ,a_{i_{\left|I\right|}}\right)$ will denote a pre-defined set of values to $x_{i}$, with the above rule requirement effectively meaning that each $x_{i}$ appearing in the second vector must equal the corresponding $a_{i}$ in the first vector. Thus the condition $x_{I}=a_{I}$ requires a specific assignment of values to $x_{i}$ for i∈I, and the rule is that if a test vector satisfies this condition, we classify it to category k.

The second sub-routine (the *construct algorithm*) builds a candidate rule R, based (initially) on a fixed training point, say a∈D, in class k. This is done by first placing all of the stubs (i,j) with non-zero counts $C_{ij}$ into a list L sorted in descending order by their values in C.

We define the candidate rule R (based on a) through the following steps. We initialize using the first stub $L_{1}=\left(i_{1},j_{1}\right)$ in the list L, together with the two fixed values $x_{i_{1}}=a_{i_{1}},x_{j_{1}}=a_{j_{1}}.$ This is the initialized form of the rule R, which requires that for any test vector, its values at the above indices $i_{1}$ and $j_{1}$ match the values of the above fixed training vector a∈D, so that $x_{i_{1}}=a_{i_{1}}$, and $x_{i_{2}}=a_{i_{2}}$. For brevity, denote the pair $\left(i_{1},j_{1}\right)=I_{1}$ and the corresponding assigned values as $\left(a_{i_{1}},a_{j_{1}}\right)=a_{I_{1}}.$

Then the content of rule R will be denoted succinctly as $R:x_{I}=a_{I}\Rightarrow classk$. Since ordering of the indices $i_{1},j_{1}$ does not matter, (as long as the indices are identified), we will henceforth write $\left(i_{1},i_{2}\right)\to \left\{i_{1},i_{2}\right\}$.

We then update rule R as follows. We find all x∈D that satisfy the initial part of rule R, i.e., $x_{I}=a_{I}$ i.e., all training points matching the two indices $\left\{i_{1},j_{1}\right\}$ of training sample a, and store them as a subcollection $D_{1}\subset D$ of the training set. We call F the fraction of data points in $D_{1}$ that have phenotype k, i.e., match the phenotype of the initial sample a∈D. When *F* is greater than or equal to a user defined *threshold*, the algorithm terminates and returns ***R***. If F>=threshold, we stop and return the current above rule R **.** If F<threshold, we continue by choosing the second stub $L_{2}=\left\{i_{2},j_{2}\right\}$ in the above list L, and augment the current rule R by adding the condition $x_{i_{2}}=a_{i_{2}},x_{j_{2}}=a_{j_{2}}$ (again written $x_{I_{2}}=a_{I_{2}}$) and maintaining the assignment of class k (i.e., the same class as the currently fixed sample a∈D). If the second stub $L_{2}$ happens to overlap with the initial stub $L_{1}$, this added condition in the rule R will clearly be consistent, being still based on the fixed sample a. We augment the current index list $I_{1}$ to a list $I_{2}$, adding to it the two new indices $i_{2}$ and $j_{2}$, so that now $I_{2}=\{i_{1},j_{1},i_{2},j_{2}$} writing the augmented rule as $R:x_{I_{2}}=a_{I_{2}}\Rightarrow classk.$ Again defining F to be the fraction of the data subset $D_{2}$ (matching the more restrictive new rule R **)** with phenotype k, we stop the algorithm and use the current rule R if F>=threshold, and otherwise augment rule R by adding the indices $L_{3}=\left(i_{3},j_{3}\right)$ to it, as above, yielding a larger set $I_{3}$ of indices and the augmented rule R: $x_{I_{3}}=a_{I_{3}}\Rightarrow classk$ , with a more restricted subset $D_{3}\subset D$, and a new value for F, now the fraction of $D_{3}$ in the class kof the fixed a∈D.This process continues until the fraction F>=threshold, e.g., 100% of the samples in D match the current set of indices, and also match the class k of the current sample a. Alternatively, the algorithm stops when all stubs in L have been exhausted.

In the examples that follow we have set *threshold* to 1. The rationale for this choice is that we allow overfit with intention of pruning the overfit rules in order to find more generalizable forms. We make this choice because from the perspective of discovery, we assume that it is more desirable to capture as much of a true underlying rule as possible and then prune back to a shorter one, than it is to extract a concise rule. In practice one might decide to tune the *threshold, F,* to approximate the overall precision of the model in order to identify less complex rules or tune it as a hyper-parameter in order to reduce the combinatorial search space.

Algorithm 2

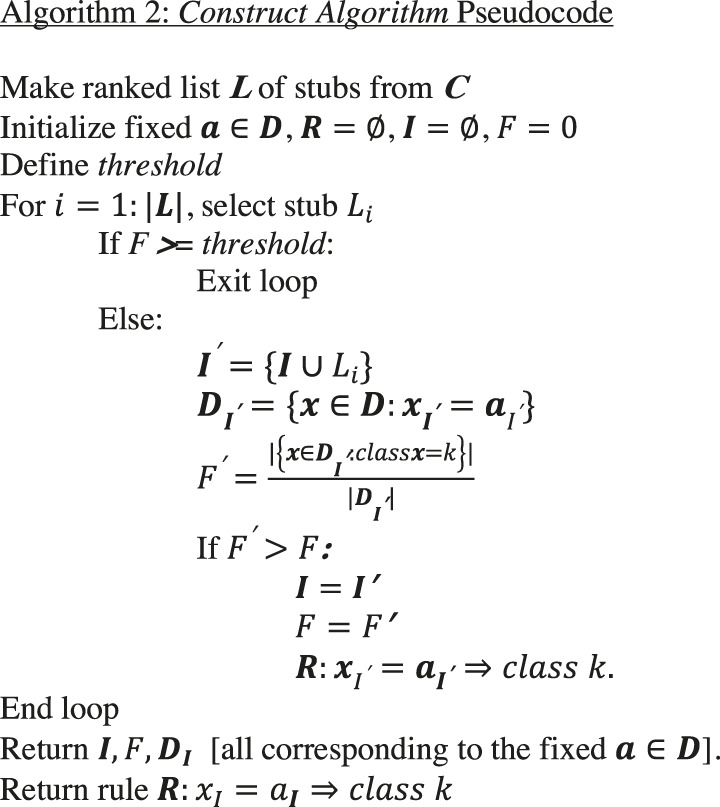

### Curating Candidate Rules

The *count* and *construct* algorithms are the heart of BowSaw. In our workflow, we apply these algorithms to each sample $a\in D^{k}$, where $D^{k}$ denotes the set of samples in dataset D with phenotype k. At this stage in the algorithm, we have associated a single candidate rule q for each vector in $a\in D^{k}$. The union of these candidate rules over all samples in D will form a list which we will denote as $Q_{k}$, which ranks each rule q by the size $\left|D_{q}\right|$ of the set $D_{q}$ consisting of all samples a∈D consistent with rule q. Since $Q_{k}$ may include many redundant rules or rules that strictly extend each other, we have another sub-routine (the *curate algorithm*) to generate a concise set of candidate rules that collectively account for all samples $D^{k}$ in class k. Briefly, we initialize a list ***H***, with the element $q_{1}\in Q_{k}$ representing the largest set $D_{q_{1}}\subset D^{k}$ of samples. At each stage, the next rule in $Q_{k}$ is selected so as to be satisfied by the largest number of elements$a\in D^{k}$ that do not satisfy any of the previous rules. This rule is then added to H, with ties resolved randomly. This is then continued until the elements in $D^{k}$ satisfying at least one rule in H are exhausted.

Algorithm 3

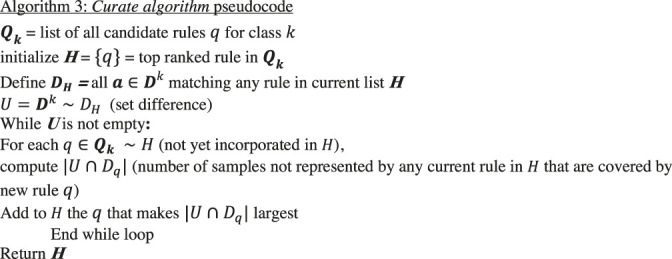

### Constructing Sub-Rules

In any given dataset, rules are rarely perfectly associated with specific phenotypes. Given the current list H of rules describing phenotype k as above, we may consider a looser set of rules by creating a new list $Q_{new}$ consisting of rules in H together with sub-rules satisfying some user defined minimal complexity criterion and precision thresholds which serve to exclude low quality rules from the analysis. Given a rule is the conjunction of a set of conditions, by sub-rule we mean the conjunction of a subset of these conditions. The list $Q_{new}$ can be treated precisely as the list $Q_{k}$ was above, resulting in a new curated list $H_{new}$ obtained as earlier, yielding a new candidate rule set which has a reduced likelihood of overfitting the data.

Thus, we will require a strategy for selecting a set of candidate sub-rules that account for all samples with desired observed phenotype class k. Candidate sub-rules are shorter candidate rules (with less complexity, likely less precise, and more broadly applicable) derived from larger candidate rules by keeping one or more (generally i) variables. For each candidate rule in ***H***, and complexity level *i*, we include only sub-rules that meet the user-defined complexity criterion, designated as complexity level i. We place each of the sub-rules derived from H at complexity level i into a new list $Q_{new}$. For each rule in $Q_{new}$ its precision is calculated with respect to the class k, and those rules with a precision below a given threshold are eliminated. Finally, this reduced list is subject to the above *Curate* algorithm again.

Algorithm 4

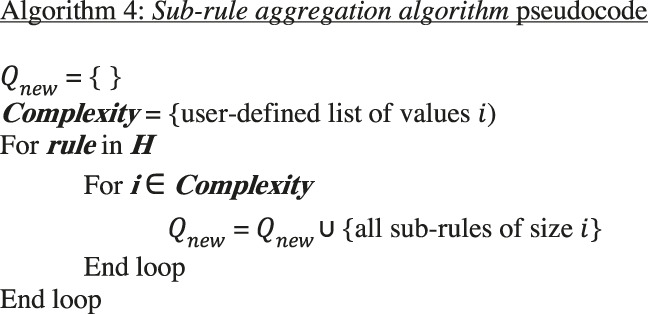

Within the above aggregation algorithm, $D_{q}$ is determined for each rule in $Q_{new}$ which is then pruned with the *curate* algorithm to produce $H_{new}$.

The algorithms described above are generalizable to multi-classification tasks but are currently limited to discretized or categorical representations of the feature space. Pseudocode for implementing each of the algorithms described above along with an implementation of the algorithms in R (R Core Team, 2020) can be found in the supplemental files and on github: https://github.com/segrelab/BowSaw.

## Results

### Application to Simulated Data

To test the capacity of BowSaw to recover multiple decision rules when the ground truth is known, we applied it to increasingly challenging simulated data sets. These data sets consist of binary vectors representing different samples. The phenotype associated with each sample is a function of the corresponding vector. The function consists of a set of multiple mutually distinct Boolean rules, such that if a rule is satisfied, it will cause the sample to have the phenotype with a certain probability (which we call here “penetrance” because of its resemblance to the genetics concept). The first dataset (IDEALIZED) we use is relatively simple and includes multiple equally prevalent rules. It is also generated under the assumption that there are no unmeasured confounders, i.e., that if a sample does have a phenotype, then it must be satisfying at least one of the above rules. We then apply BowSaw to a more challenging scenario (INTERMEDIATE) in which the phenotype-generating rules differ in their relative prevalence and the assumption of unmeasured confounders is violated. Finally, is a set of data sets with complex co-varying parameters (COMPLEX), we systematically varied the underlying parameters of the simulation and examined the relationship between summary statistics of the RF performance and the ability of BowSaw to generate candidate rules containing the true phenotype-generating rules.

**FIGURE 2:**
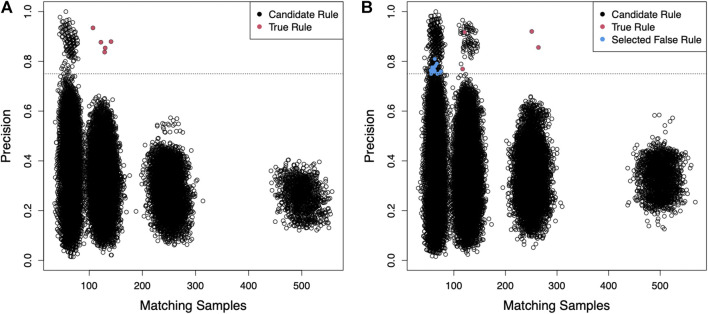
For both scenarios 2,000 samples were generated with 100 randomly generated binary features. **(A)** The generality of sub-rules (number of points that exactly satisfy the rule criteria) is plotted against their precision for the IDEALIZED scenario (Five rules that cause the phenotype and no noise). Each point represents a unique sub-rule. *X*-axis is the number of samples in the dataset that exactly match the pattern defined by the rule. *Y*-axis is the fraction of matching samples with the observed phenotype (i.e., precision of the rule). Each cluster of points corresponds to decreasing rule complexity from 5 variables per rule to 2 on the right-most cluster. These clusters appear because the values of each variable are produced by an identical binomial distribution. The dashed line is the precision threshold we chose in order to exclude low quality rules. Only candidate rules with precision above this threshold were considered for the curate algorithm. Red points are the causative sub-rules we defined. BowSaw correctly identified all five red points in this scenario. **(B)** Candidate sub-rules generated for the more challenging INTERMEDIATE scenario. We defined 5 causative rules of varying lengths in this scenario and allowed 2% of samples without a causative rule to be assigned the label. BowSaw completely recovered 4 of the causative rules (red points). The longest rule which involved 5 variables was not fully recovered by any candidate rule. Rules that were selected by the Curate algorithm because of their contribution to additional coverage but that did not contain a complete true rule are indicated by blue points.

For the IDEALIZED scenario, we simulated a data set of 100 independent and identically distributed random binary variables and 2,000 samples. We randomly defined five rules, each requiring four randomly selected variables to have specific values (e.g., all variables equal to 1) in order to assign a hypothetical phenotype with likelihood between 0.8 and 0.9. Here we present the results of this scenario with a specified random seed, but other seeds and parameters can be explored using the scripts provided in the supplemental files. Using these parameters, 497 samples were assigned the phenotype and BowSaw produced a set of 135 unique candidate rules ranging in complexity from six to fourteen variables. From these rules, we produced all sub-rules involving anywhere between two and five variables, which resulted in unique 50,034 sub-rules. To reduce the number of sub-rules that the *Curate* algorithm would need to examine, we eliminated from consideration any rules that had a class precision below 80%. We selected an 80% threshold because in the cluster centered around 125 matching samples there is a small cloud of rules that are clearly segregating the phenotype more efficiently than the others (Figure 2A). We selected the most general remaining sub-rule to initialize our list of candidate rules. This produced a final list consisting of five candidate rules that accounted for all of the samples with the phenotype and were each one of the true phenotype generating rules (Figure 2A red points). These results demonstrate that in an ideal scenario with no measurement errors, BowSaw is indeed capable of recovering multiple true rules.

For the more challenging scenario (INTERMEDIATE), we generated the data set as before, except that this time we allowed the five underlying rules to vary in complexity from three to five variables. Varying the complexity of rules resulted in different prevalence among them, as rules that are more complicated are less likely to appear in the data. In this case, we had one rule of complexity five, two that required four variables, and two that used three variables. We also added background noise by randomly assigning the phenotype to 2% of samples that did not possess any of the rules, 655 samples were assigned the phenotype. BowSaw produced 176 unique candidate rules involving between six to thirteen variables. From this list we generated 68,938 sub-rules and chose a precision threshold of 75% because there are two clusters at ∼|***T***| = 125 that begin to clearly separate in that range and the two outlier points at ∼|***T***| = 250 do not combine to account for all of the phenotype (Figure 2B). Applying the *Curate* algorithm to the rules meeting this threshold selected 19 candidate sub-rules, the top four (when ranked by |***T***|) of which were true rules (red points). The remaining 15 rules were noise rules (blue points). The rule of five variables was not recovered. These results show that BowSaw is able to recover strongly associated patterns (and in this case, causal patterns) even in the presence of noise, but low prevalence rules can be masked by more highly prevalent rules.

We used the same data generation method to investigate BowSaw’s ability to produce candidate rules containing true rules when the underlying parameters change. We applied BowSaw to 20,000 simulated data sets where we randomly altered the number of features (50–1,000), sample size (200 or 2,000 samples), complexity of the rules (2–8 variables), number of rules (2–8), the likelihood of each rule assigning the phenotype (0.0005–1), and the background noise (1x10^−5^ to 0.1). For each simulation we extracted a single candidate rule per sample with the assigned phenotype and ranked them without generating sub-rules.

To investigate how effectively BowSaw recovers true rules, for each simulation we calculated the fraction of true rules fully recovered, the probability of fully recovering at least one rule, the median rank of the first recovered rule when at least one is recovered, and the mean rule completeness of recovered rules. We investigated the relationship of these measurements to the to the ROC-AUC, PR-AUC, number of features, and sample size. These values were chosen because they are easily accessible to researchers during model building and could potentially be used to assess the likelihood of obtaining useful insights from applications of BowSaw.

ROC-AUC, PR-AUC, and sample size are positively correlated with full recovery of true rules, mean completeness of recovered rules, and median rank. Number of features was negatively correlated with these values. These correlations are summarized in Table 1. The probability of recovering at least one true rule gradually decreases with increasing feature space, gradually increases with increasing sample size, and forms a sigmoidal curve with both ROC-AUC and PR-AUC. Plots depicting the relationship of the four metrics with the fraction of fully recovered rules, probability of recovering at least one rule, median rank of rules, and mean rule completeness can be found in Supplementary Figures S1–S4.

**TABLE 1:** Correlation of performance metrics and data dimensions with rule recovery.

	ROC-AUC	PR-AUC	N Features	Sample size
Fraction of rules recovered	0.672	0.585	-0.151	0.556
Mean partial recovery all rules	0.683	0.581	-0.251	0.657
Median rank of first recovered rule	0.268	0.195	-0.073	0.071

### Application to Human Microbiome Data

Irregular distributions of microbial taxa within the gut are often associated with serious illnesses such as Crohn’s disease or ulcerative colitis (Carding et al., 2015; Levy et al., 2017). Human microbiome studies regularly use 16s rRNA amplicon sequencing methods and extensive reference databases to report on microbial taxa found in samples as operational taxon units (OTUs). RF classifiers are frequently built using counts of OTUs to accurately discriminate between disease and healthy patient samples (Ai et al., 2019; Vangay et al., 2019). Despite their demonstrated effectiveness as good classifiers of Crohn’s disease, studies that look to discover associations with disease status typically focus on individual OTUs, while specific microbial association rules found by RF are not discussed, as a result it is uncertain how heterogeneous study cohorts are. To investigate potential rule heterogeneity in a human microbiome cohort we downloaded processed files from the Human Microbiome Project for inflammatory bowel disease (IBD) (Proctor et al., 2019) which contain information on the taxonomic profiles of 982 OTUs in 178 patients–86 of which have been diagnosed with Crohn’s disease, 46 diagnosed with ulcerative colitis, and 46 diagnosed as non-IBD. We were specifically interested in finding rules that separate the Crohn’s disease samples from ulcerative colitis and non-IBD, so we framed the problem as a binary classification task with Crohn’s disease as the target phenotype.

**FIGURE 3:**
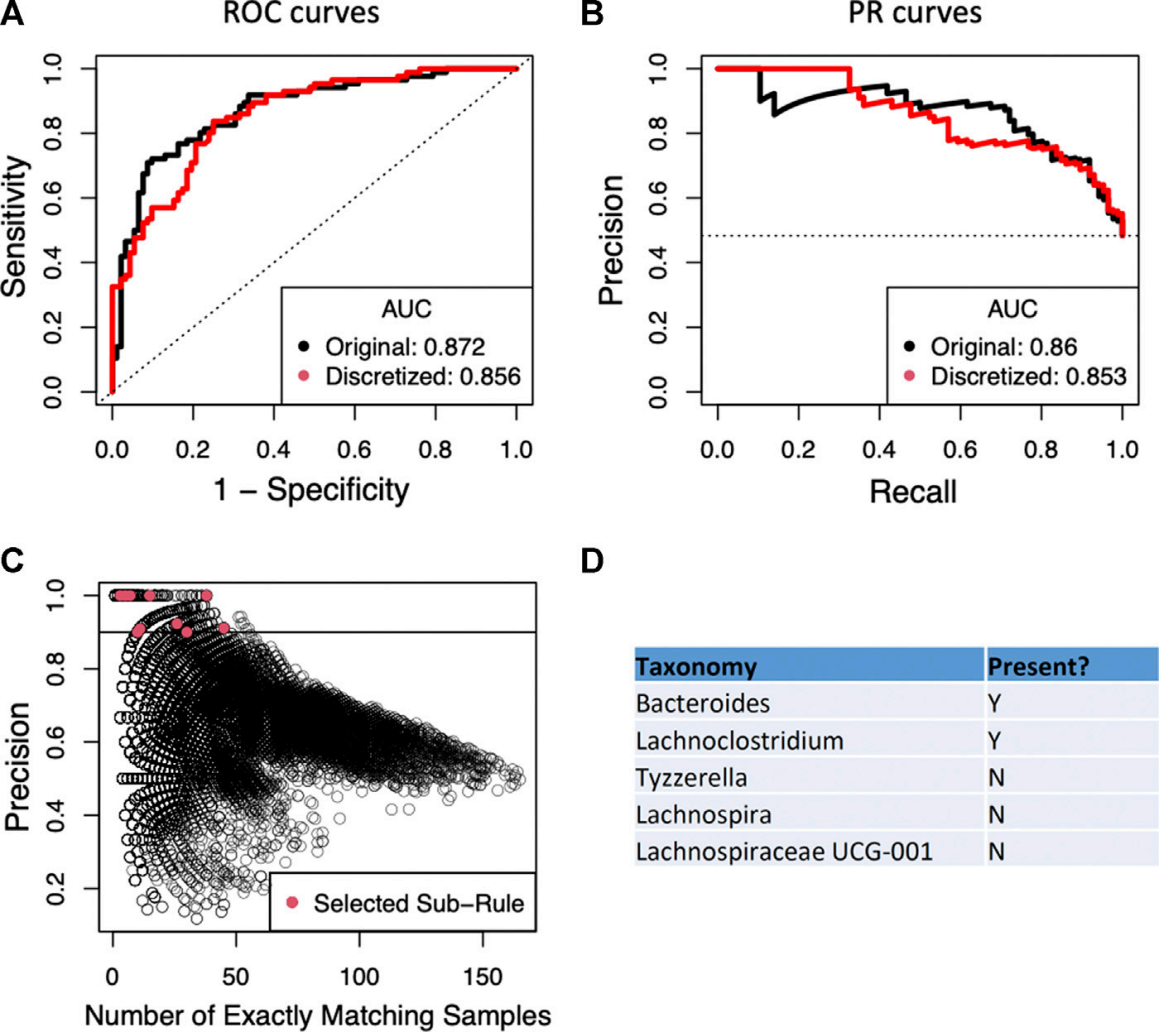
**(A)** Performance of the random forest classifier as measured by area under the receiver operator curve (ROC-AUC) is not strongly perturbed by simplifying OTU representation to a presence/absence scheme vs. the original continuous count. Dashed line indicates the performance of a perfectly random classifier. **(B)** The area under the curve of the precision recall curve is similarly not strongly affected by the new representation scheme. Dashed horizontal line is the random performance line. **(C)** Each point represents a unique candidate sub-rule. On the *x*-axis is the number of samples in the data matrix that are subject to that rule. The *y*-axis represents what fraction of matching samples were diagnosed as Crohn’s disease. **(D)** The taxon identities of the OTUs that make up the most generally applicable of the sub-rules where all matching samples have the Crohn’s disease label.

Since the current implementation of BowSaw is limited to finding rules when the variables have categorical values, we first converted the OTU counts of each taxon to a simple presence/absence scheme. This resulted in nearly equivalent RF performance relative to training RF with the original continuous OTU inputs: ROC AUC of 0.856 (binary) vs 0.872 (continuous) and PR AUC of 0.853 (binary) vs 0.86 (continuous) (Figures 3A,B). This is an important result because it allows us to think about associations just in terms of presence or absence of an OTU without sacrificing much in model performance. We next applied BowSaw to the Crohn’s disease samples and generated 86 unique classification rules. These rules ranged in complexity from 4 OTUs to 16 OTUs (median 9 OTUs) and applied to as few as 1 sample up to 36 samples (mean 6.3, +/-6.6, median: 4). The most broadly applicable rule involved 8 OTUs.

We then applied the Sub-rule algorithm and visualized 56,902 resultant sub-rules ranging in complexity from 2 to 7 variables (Figure 3C). There were 1,941 sub-rules with precision = 1. We selected the most general of these rules (max*|* ***T*** *|*) to be the top candidate for the curate algorithm and found that it considers the status of 5 OTUs and accounts for 38 of the 86 Crohn’s disease samples (Figure 3C), this rule was derived from the rule that considered the status of 8 OTUs and accounted for 36 Crohn’s disease samples. We set a precision threshold of 90% and ended up with 10 sub-rules involving an average of 4 OTUs (min = 2, max = 7), each derived from a unique parent rule (average OTUs = 9.6, min = 6, max = 16), that together account for all 86 Crohn’s disease samples and an additional 11 non-Crohn’s disease samples (4 non-IBD, 7 ulcerative colitis). The top five rules combine to account for 78 of 86 Crohn’s disease samples and include 10 non-Crohn’s disease samples (Table 2).

**TABLE 2:** Association rules identified by BowSaw that account for all Crohn’s disease samples.

Rule	CD samples	Non CD samples	New samples covered	Taxonomy	Presence
1	38	0	38	*Bacteroides (genus)*	y
				*Lachnoclsotridium (genus)*	y
				*Tyzzerella (genus)*	n
				*Lachnospira (genus)*	n
				*Lachnospiricae UCG-001 (genus)*	n
2	41	4	20	*Dialister (genus)*	y
				*Christensenellacea R7 group (genus)*	n
				*Collinsella (genus)*	n
				*Ruminococcaceae (family)*	n
				*Finegoldia (genus)*	n
				*Ruminococcus (genus)*	n
3	9	1	9	*Ruminococcus (genus)*	y
				*Ruminococcaceae UCG-002 (genus)*	n
				*Lachnospirceae (family)*	n
4	24	2	6	*Streptococcus (genus)*	y
				*Tyzzerella (genus)*	n
				*Lachnospiraceae (family)*	n
				*Hafnia obesumbacterium*	n
5	27	3	5	*Lachnospiricae UCG-008 (genus)*	y
				*Ruminococcus 1 (genus)*	n
				*Eubacterium eligens group*	n
6	5	0	2	*Ruminococcus 1 (genus)*	y
				*Dorea (genus)*	n
7	7	0	2	*Bacteroides (genus)*	y
				*Dialister (genus)*	n
				*Eubacterium rectale group*	n
8	15	0	2	*Lachnospiraceae NK4A136 group*	y
				*Eubacterium eligens group*	y
				*Tyzzerella (genus)*	n
				*Christensenellacea R7 group (genus)*	n
				*Lachnospira (genus)*	n
9	3	0	1	*Ruminococcus gnavus group*	y
				*Veillonella (genus)*	n
				*Bacteroides (genus)*	n
				*Finegoldia (genus)*	n
10	10	1	1	*Parabacteroides (genus)*	y
				*Eubacterium eligens group*	y
				*Ruminococcaceae Ucg-003 (genus)*	n

The top candidate rule is comprised of the presence of *Bacteroides* and *Lachnoclostridium* and the absence of three genera from the family *Lachnospiraceae: Lachnospira*, *Tyzerrella,* and *Lachnospiracea UCG 001* (Figure 3D). Detection of *Bacteroides* was nearly ubiquitous within the cohort, it was found in 170 of 178 total samples, but only 3 of the samples in which it was missing are diagnosed as Crohn’s disease. For the remaining taxa we performed a t-test comparing the distribution of the taxa in Crohn’s disease vs. ulcerative colitis and vs. healthy samples. *Lachnoclostridium* was frequently found in Crohn’s disease (67/86) but not in ulcerative colitis (27/46, *p* = 0.02) and was detected at roughly the same rate in non-IBD samples (34/46, *p* = 0.616). Detection of *Lachnospira* was depleted in Crohn’s disease samples (20/86) relative to ulcerative colitis (20/46, *p* = 0.022) and to non-IBD samples (31/46, *p* = 9.9–7). *Tyzzerella* was also detected at a lower rate in Crohn’s disease (63/86) relative to ulcerative colitis (24/46, *p* = 0.019) and non-IBD (24/46, *p* = 0.019). *Lachnospiracea UCG 001* was rarely detected in Crohn’s disease (4/86) which is a lower rate than it was detected in ulcerative colitis (9/46, *p* = 0.022) and in non-IBD samples (19/46, *p* = 1.45–5).

### Application to Mushroom Data

To further demonstrate the generalizability of our approach to non-binarized datasets we identified the mushroom data set from the UCI machine learning repository (UCI Machine Learning Repository). This data set contains 8,123 observations of poisonous (3,915) and edible (4,208) mushrooms. There are 22 categorical features ranging from 2 to 12 categories. The two classes are perfectly separable, and the documentation accompanying the matrix describes a set of rules that separate all edible mushrooms from poisonous samples. This rule set provides a good baseline to compare the complexity of the final rule sets obtained with BowSaw to.

We applied our approach to the original matrix of 22 features with multiple categories and to a binarized transformation where we give each category its own column (117 features). In both cases we used BowSaw to extract classification rules that account either for all edible mushrooms or for all poisonous mushrooms. Since the samples are fully separable we again set *F* = 1. This setting resulted in candidate rules ranging in complexity from 2 to 9 variables. We examined all sub-rules from complexity 1 up to complexity 9 and retained only those that were entirely associated with the target class (precision = 1) for curating a short list. In total we generated 4 different rule lists that fully separate edible from poisonous mushrooms and also differ from the data donor’s contributed list. Each list is composed of 7 rules. The rule lists obtained from each run are described in Supplementary Table S1 along with the contributed list

## Discussion

Linear models for classification such as logistic regression are often the “go to” approach due to their ease of implementation and interpretation of coefficients. However, many biological datasets contain non-linear interactions between features. In these situations it is not uncommon for random forests to significantly outperform logistic regression. Interpretation of random forest models for classification is not straightforward and may be complicated when there are multiple rules (combinations of variables and their specific values) associated with a phenotype of interest. Our newly developed BowSaw approach, best applied when random forest is the appropriate classifier, is an algorithmic method for identifying the rules that a trained random forest model uses to make classifications when the values are categorical in nature. By taking advantage of the structure of trees found within a random forest, BowSaw produces a set of multiple decision rules that combine to account for each sample with a given observed phenotype. When the variables are the presumed causal agents, these rules represent plausible mechanistic relationships.

Results on simulated data demonstrate that when there are multiple rules associated with a single phenotype label that BowSaw is capable of faithfully identifying them. Application to data from the human microbiome project offers further evidence that BowSaw provides an efficient way of generating plausible hypotheses for high throughput metagenomics studies. In particular we identified a rule that utilizes a presence/absence pattern of five microbial taxa (present: *Bacteroides, Lachnoclostridium*; absent: *Lachnospira, Lachnospiracea, Tyzerrella*) that accounts for nearly half of all Crohn’s disease samples in the cohort (38/86). This specific pattern of microbial colonization in the guts of Crohn’s disease patients is unreported, but each taxon’s respective enrichment or depletion status and association with disease status has been reported. If the cohort of patients in the human microbiome study are representative of all people afflicted by Crohn’s disease, then this rule represents a significantly large subset of those suffering. Inquiries into the relationship of the taxa included in this rule with disease status may yield important insights into the mechanisms of the disease and potential therapeutic strategies for this sub-population. Of the five associated taxa, we suspect that the absence of *Lachnospira, Lachnospiracea UCG 001,* and *Tyzzerella* are biologically meaningful. We have reason to believe so because it has been reported that the *Lachnospiraceae* family is generally suppressed in Crohn’s disease (Loh and Blaut, 2012; Geirnaert et al., 2017; Nagao-Kitamoto and Kamada, 2017). *Lachnospira* has been reported as depleted with respect to Crohn’s disease several times (Wright et al., 2017; Wang Y. et al., 2018). The depletion of *Tyzzerella* has been associated with chronic intestinal inflammation and supplementation suggested as a probiotic for Crohn’s disease (Berry et al., 2018; Chen et al., 2018). While the relationship of *Lachnospiracea UCG 001* with Crohn’s disease is still unclear, its depletion has been reported in mice displaying symptoms of anhedonia and it was significantly enriched in anhedonia resilient mice (Yang et al., 2019). Partly because IBD is frequently accompanied by depression, anhedonia has been suggested as an important symptom in the diagnosis of IBD (Carpinelli et al., 2019). The associations of the individual OTUs defined by this rule are consistent with previously reported findings in the existing literature and describe a taxonomic profile that exclusively identifies a large sub-population of Crohn’s disease samples within this cohort. The presence of *Bacteroides* does not appear to be particularly useful and in this context is probably preserved because it causes a perfect association, although high levels of some species are implicated in the pathology of Crohn’s disease (Rabizadeh et al., 2007). *Lachnoclostridium* is differentially distributed across the three classes. Notably it is less frequently detected in ulcerative colitis relative to Crohn’s and non-IBD samples, which roughly resemble one another. Increased levels of this genus were detected in rats that showed relief of colitis symptoms after treatment with a proposed therapeutic agent (Wang K. et al., 2018).

The current implementation of the algorithms is restricted to classification tasks with categorical predictor values. This is a challenge that can be addressed in future variants of this approach, in order to make it more generally applicable. Future work could also focus on extending these approaches to the interpretation of regression models or to consider the effect of counting stubs of higher-order interactions or co-occurring pairs on bookkeeping and rule extraction as opposed to strict parent-child relationships. We anticipate that the concept at the core of BowSaw and its different possible extensions could help uncover complex feature-phenotype maps for other types of biological datasets.

## Data Availability

Data and code presented in this study are available on GitHub (https://github.com/segrelab/BowSaw). Additional analyses are included in the article/Supplementary Material.
