# Supplementary material for: BowSaw: Inferring Higher-Order Trait Interactions Associated With Complex Biological Phenotypes
Source: Front Mol Biosci. 2021 Jun 17;8:663532. doi: 10.3389/fmolb.2021.663532 (PMC8245782; doi:10.3389/fmolb.2021.663532)
Supplement: Supplementary file 1 [file DataSheet1.PDF]

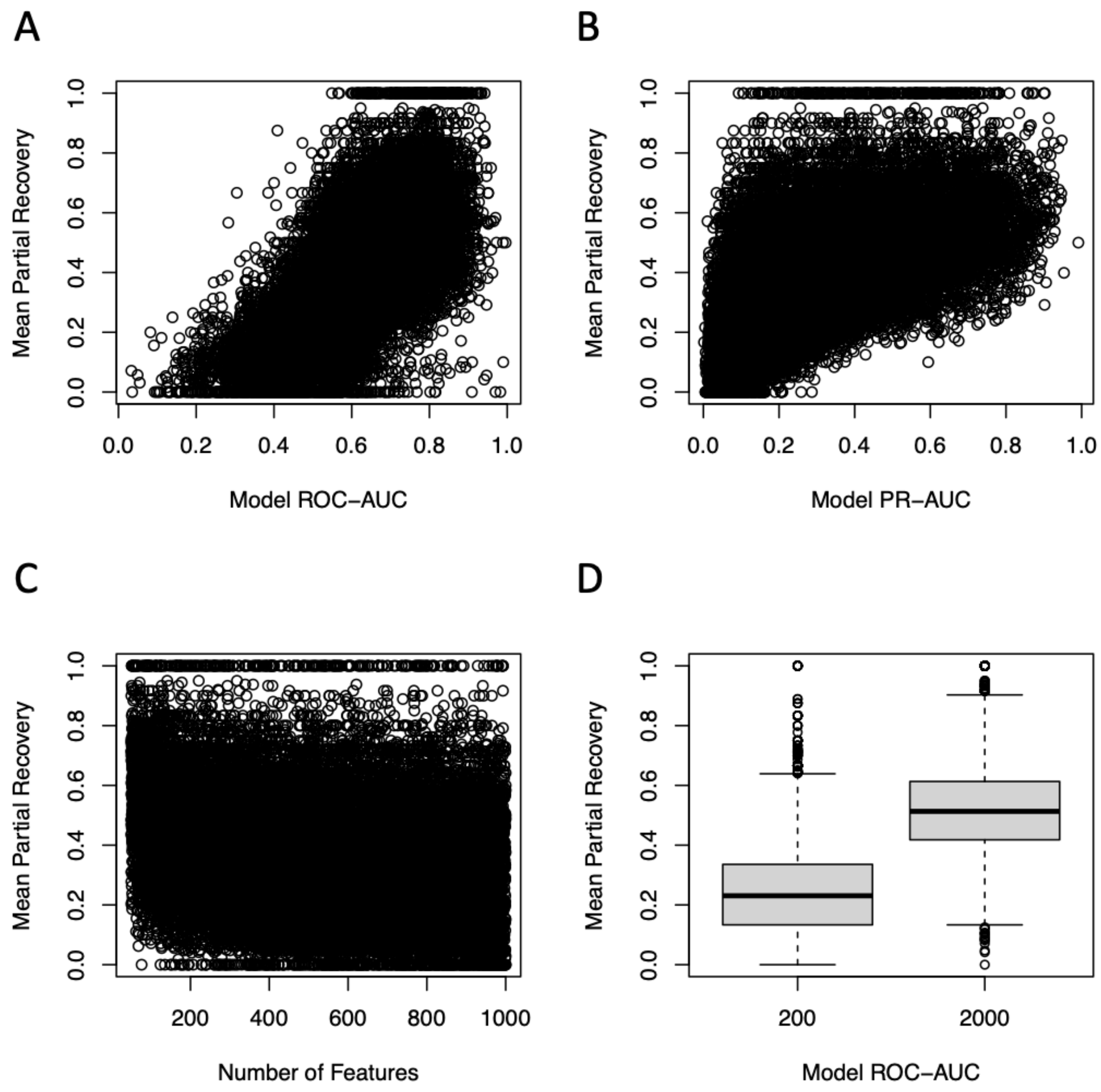

Figure S1. Mean Partial Recovery of True Rules, when a true rule is recovered by at least one candidate rule, what is the mean fraction of the correct variables recovered? **A.** Mean recovery against ROC-AUC. **B.** Mean recovery against PR-AUC. **C.** Mean recovery against features used **D.** Mean recovery against sample size.

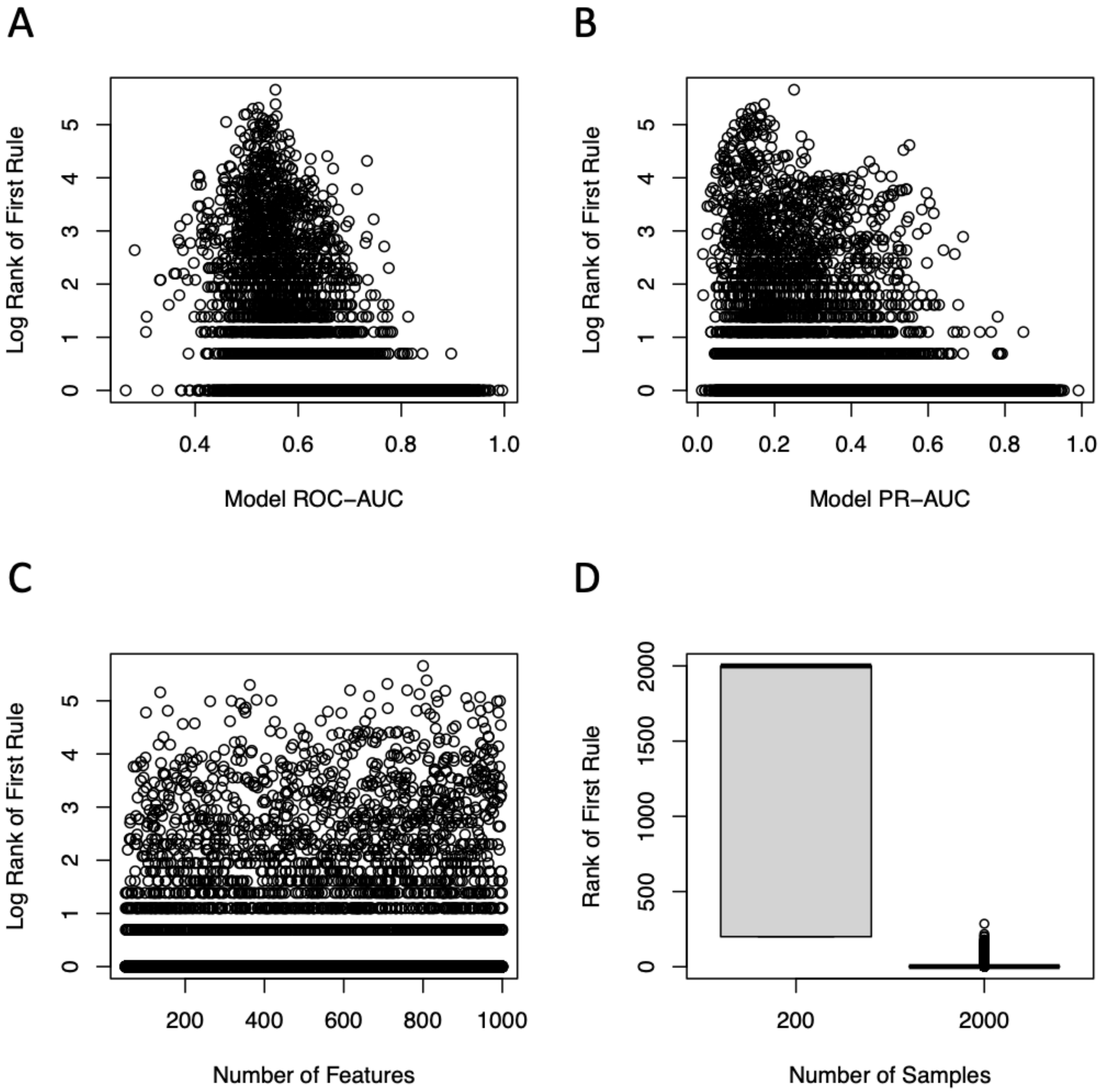

Figure S2. Rank of the first candidate rule when at least one true rule is recovered. **A.** Log rank against ROC-AUC. **B.** Log rank against PR-AUC. **C.** Log rank against features used **D.** Log rank against sample size.

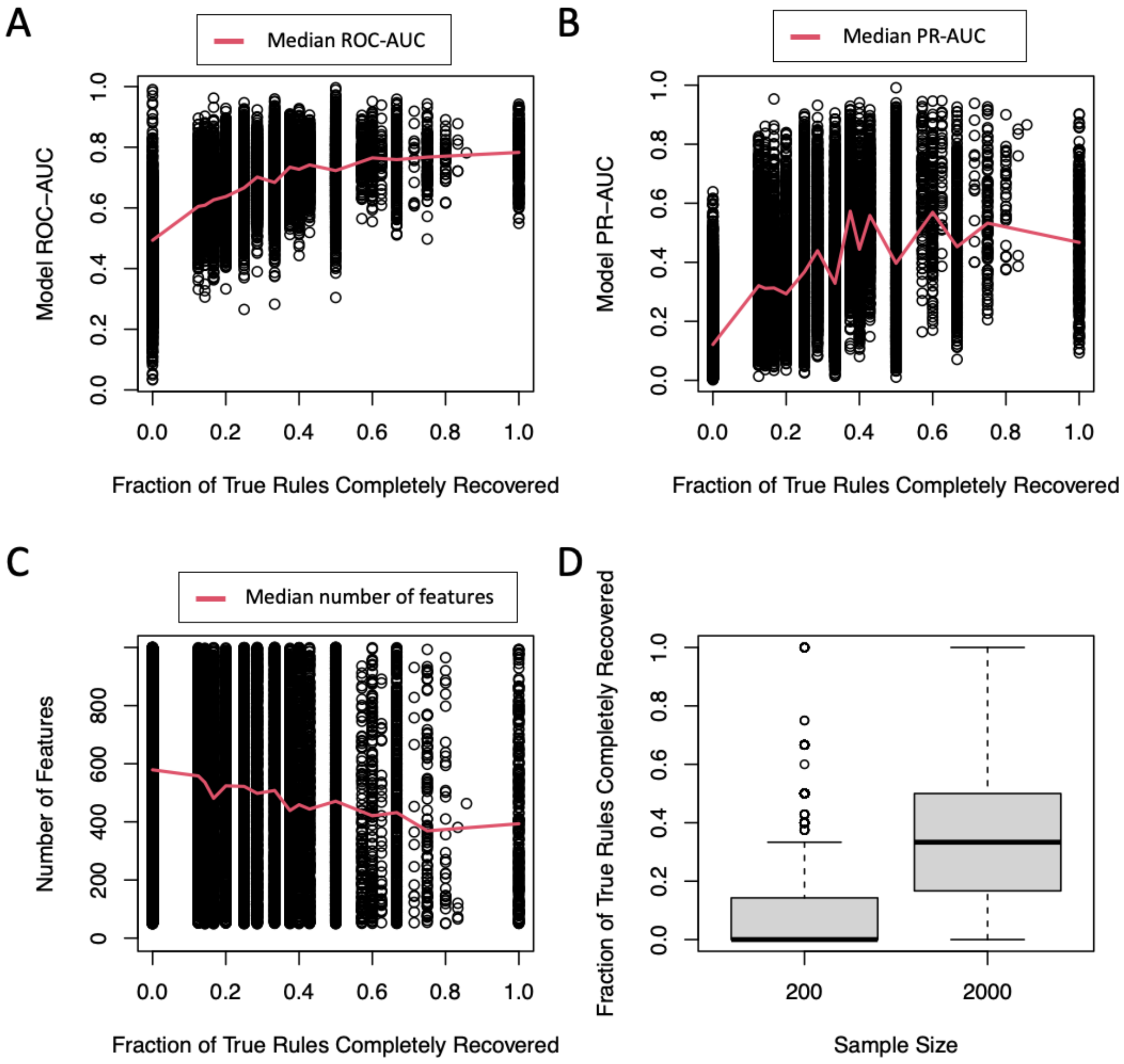

Figure S3. What fraction of true rules are completely recovered by BowSaw? **A.** Instances with higher rates of recovery tend to be associated with increasing ROC-AUC metrics, although variance is high. **B.** PR-AUC against fraction of true rule recovery. **C.** Feature space against fraction of true rule recovery. **D.** Sample size against fraction of true rules recovered.

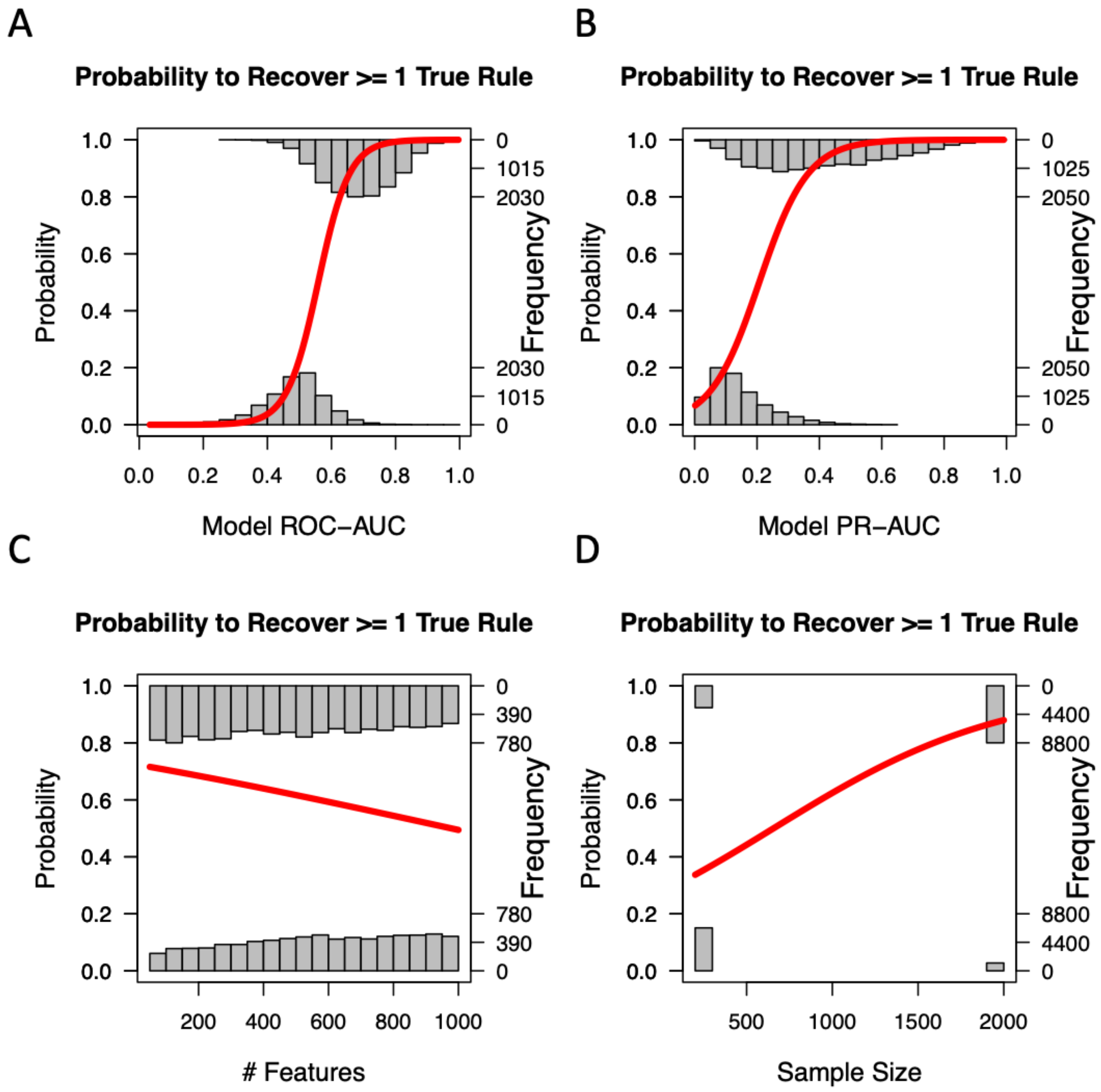

Figure S4. What is the probability of recovering at least one true rule completey? **A.** Probability as a function of ROC-AUC. **B.** Probability as a function of PR-AUC. **C.** Probability as a function of feature space. **D.** Probability as a function of sample size.
